# Identification and Investigation of the Genetic Variations and Candidate Genes Responsible for Seed Weight via GWAS in Paper Mulberry

**DOI:** 10.3390/ijms232012520

**Published:** 2022-10-19

**Authors:** Yanmin Hu, Xianjun Peng, Shihua Shen

**Affiliations:** Key Laboratory of Plant Resources, Institute of Botany, The Chinese Academy of Sciences, Beijing 100093, China

**Keywords:** genome-wide association study, seed weight, woody plant, genetic variation, candidate gene, fatty acid desaturase

## Abstract

Seeds directly determine the survival and population size of woody plants, but the genetic basis of seed weight in woody plants remain poorly explored. To identify genetic variations and candidate genes responsible for seed weight in natural woody populations, we investigated the hundred-seed weight of 198 paper mulberry individuals from different areas. Our results showed that the hundred-seed weight of paper mulberry was significantly associated with the bioclimatic variables of sampling sites, which increased from south to north along the latitudinal-temperature gradient. Using 2,414,978 high-quality SNPs from re-sequencing data, the genome-wide association analysis of the hundred-seed weight was performed under three models, which identified 148, 19 and 12 associated genes, respectively. Among them, 25 candidate genes were directly hit by the significant SNPs, including the WRKY transcription factor, fatty acid desaturase, F-box protein, etc. Most importantly, we identified three crucial genetic variations in the coding regions of candidate genes (*Bp02g2123*, *Bp01g3291* and *Bp10g1642*), and significant differences in the hundred-seed weight were detected among the individuals carrying different genotypes. Further analysis revealed that *Bp02g2123* encoding a fatty acid desaturase (FAD) might be a key factor affecting the seed weight and local climate adaptation of woody plants. Furthermore, the genome-wide investigation and expression analysis of *FAD* genes were performed, and the results suggested that *BpFADs* widely expressed in various tissues and responded to multiple phytohormone and stress treatments. Overall, our study identifies valuable genetic variations and candidate genes, and provides a better understanding of the genetic basis of seed weight in woody plants.

## 1. Introduction

Seeds play important roles in the plant’s life cycle, especially perennial woody plants, which directly determine the population size and continuation [1,2]. The growth environmental conditions of plants, such as temperature, precipitation, illumination and soil, will affect the nutritional reserves of seeds [3], which also help plants adapt to changeable environments [4]. Studies have shown that variation in seed size is important for seed dormancy [5], seed dispersal [6], stress tolerance, etc. [7]. Under the double pressure of genetic evolution and natural selection, plants have adapted to the local climate to produce different sizes of seeds [8]. Previous studies have revealed that the seed weight within the same species significantly increases at higher latitudes [9], while other studies find that plants from lower latitudes have higher seed weight [10], which indicates that the effects of the growing environments on seed weight are different among species [11]. Furthermore, previous studies have also found that the seed size and fatty acids of plants increase from low latitude to high latitude [11], and the seedlings from heavier seeds will have better frost tolerance [12,13]. The distribution of woody plants from high-latitude temperate zones is limited by the adaptability of their seeds, and the sufficient nutriment is conducive to the safe overwintering of seeds [11]. Therefore, detecting genetic variations and candidate genes responsible for seed weight in natural woody populations will assist in revealing how woody plants cope with variable climates.

Meanwhile, the seed weight and size also determine the crop yield, and the studies in different crops have identified some important genes associated with seed development [14]. *GS2* (*Grain Size 2*) encodes a growth-regulating factor, and the overexpression of *GS2* can enhance the grain weight and yield of rice [15]. The *GW2* (*Grain Weight 2*) of rice is involved in negatively regulating the grain weight, size, and yield, which encodes a E3 ubiquitin ligase [16]. In addition, *OsPPKL1* [17], *TGW6* [18], *GW6a* [19] and *OsmiR396a* [20] also play important roles in regulating the grain size and weight of rice. In soybean, *PP2C-1* and *GmCYP78A5* could increase the seed weight and seed size of transgenic plants [21,22]. In woody plants, transgenic experiments have proved that the overexpression of *Auxin Response Factor 19* could increase the seed size and yield of *Jatropha curcas* [23]. Furthermore, a previous study indicates that *LcCWIN5* and cell wall invertase are involved in regulating the seed development and seed size in litchi (*Litchi chinensis* Sonn.) [24]. However, the genetic basis of seed weight is still poorly understood in woody plants, and it is necessary to detect the related genes regulating the seed weight or size in woody plants, especially in natural woody populations.

In recent years, the genome-wide association study (GWAS) has been used to detect the candidate genes regulating seed size and weight in model plants and crops. For example, the GWAS of seed size detected 38 significant loci in *Arabidopsis thaliana* and the natural variations in the associated gene *CYCB1;4* (*cyclin B1;4*) significantly influence the seed size [25]. Based on 996,722 SNPs from 270 rice accessions, a GWAS analysis of seed size identified a novel gene, *OsSNB*, and the knockout mutant plants had higher grain weight and size [26]. The association analysis of seed weight in soybean has identified candidate genes encoding the AP2 transcription factor and histidine phosphotransfer protein, which also affects the seed weight and seed size in other species [27]. Furthermore, using SNPs from 197 watermelon accessions, genome-wide association analyses on the seed weight and seed size traits were performed and 11 significant SNPs were identified, and the candidate genes might be involved in the abscisic acid metabolic pathway [28]. The GWAS of seed weight and seed size detected 17 loci and six candidate genes in cowpea, and the regions associated with the seed size variations were identified [29]. In addition, a GWAS analysis of seed weight was also performed in peanut, which identified significant SNPs and potential candidate genes [30]. 

Paper mulberry (*Broussonetia papyrifera*), a perennial and dioecious woody plant from the Moraceae family, is normally used in papermaking, livestock breeding, medicine, etc., which has a wide range of temperature adaptability [31]. The seed of paper mulberry is named *Fructus Broussonetiae*, which has important economic values with a long history of the medicine application [32]. To identify genetic variations and candidate genes regulating the seed weight of woody plants, we collected the seeds of paper mulberry from different areas in China and analyzed the relationships between the hundred-seed weight and bioclimatic variables of sampling sites in this study. The genome-wide association analysis of hundred-seed weight was performed and important candidate genes were investigated, which provides significant insights into the research of seed weight in woody plants.

## 2. Results

### 2.1. Natural Variation of Seed Weight in Paper Mulberry

**Figure 1:**
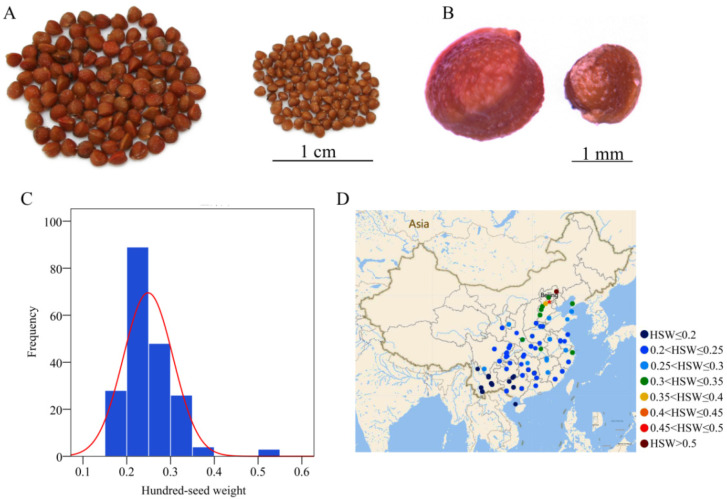
The seed size and distribution of the hundred-seed weight of paper mulberry. (**A**) One hundred seeds of paper mulberry individuals from different areas. The seeds on the left were collected from Chengde, Hebei, China (41° N), and the seeds on the right were collected from Dongfang, Hainan, China (19° N). (**B**) The seed sizes of paper mulberry individuals from different areas. The collection areas are the same as subfigure **A**. (**C**) The frequency distribution of the hundred-seed weight of paper mulberry. (**D**) The geographic distribution of the hundred-seed weight. The graded color scale from blue to red was used to display the hundred-seed weight from small to large.

To explore the genetic basis of seed weight in woody plants, we investigated the hundred-seed weight of 198 wild paper mulberry individuals which were collected from different areas (Table S1). We found that the hundred-seed weight was obviously different among paper mulberry individuals, which ranged from 0.15 g to 0.52 g (Figure 1). The results showed that the paper mulberry individuals from high latitudes had higher hundred-seed weight than the individuals from low latitudes (Figure 1D). The paper mulberry individuals having the largest hundred-seed weight were collected from Chengde, Hebei, China (41° N), and the paper mulberry individuals with the smallest hundred-seed weight were collected from Dongfang, Hainan, China (19° N) (Figure 1). 

**Figure 2:**
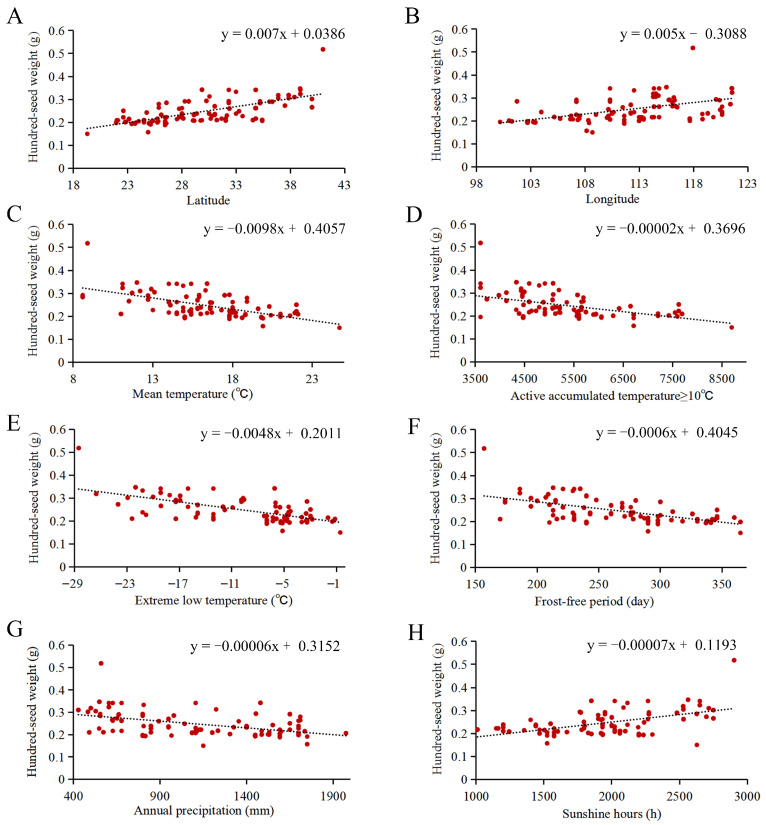
The linear fitting of the hundred-seed weight and bioclimatic variables of sampling sites. (**A**) Latitude. (**B**) Longitude. (**C**) Mean temperature. (**D**) Active accumulated temperature ≥10 °C. (**E**) Extreme low temperature. (**F**) Frost-free period. (**G**) Annual precipitation. (**H**) Sunshine hours.

In this study, the bioclimatic variables of the sampling sites also were collected (Table S2), and the linear fitting analyses of the hundred-seed weight and bioclimatic variables were performed (Figure 2). We found that the hundred-seed weight was closely related to the bioclimatic variables of sampling sites in paper mulberry, such as the mean temperature, extreme low temperature, active accumulated temperature, frost-free period, annual precipitation and sunshine hours (Figure 2). The hundred-seed weight had positive relationships with the latitude, longitude and sunshine hours (Figure 2A,B,H). For the latitude, the hundred-seed weight increased from the low latitude to high latitude along the latitudinal gradient (Figure 2A). There were negative relationships between the hundred-seed weight and the mean temperature, active accumulated temperature, extreme low temperature, frost-free period and annual precipitation (Figure 2C–G), which reflected that the hundred-seed weight of paper mulberry decreased along the temperature gradient. The above results were also supported by the paired correlation coefficients between the hundred-seed weight and bioclimatic variables of the sampling sites (Table S3). The latitude, longitude and sunshine hours of the sampling sites were significantly and positively correlated with the hundred-seed weight (*r* = 0.70 **, 0.53 ** and 0.54 **, respectively), while the mean temperature, active accumulated temperature, extreme low temperature, frost-free period and annual precipitation of the sampling sites were significantly and negatively correlated with the hundred-seed weight of paper mulberry (*r* = −0.59 **, −0.55 **, −0.64 **, −0.59 ** and −0.47 **, respectively).

### 2.2. Genome-Wide Association Study of Hundred-Seed Weight

To identify the genetic variations and candidate genes regulating seed weight in woody plants, the whole genome re-sequencing of 198 paper mulberry individuals collected from different areas was performed. Next, 614 Gb clean sequence data were generated, and the average depth was 8.2× (Table S4). After mapping to the paper mulberry reference genome and filtering, a total of 2,414,978 high-quality SNPs were detected. 

**Figure 3:**
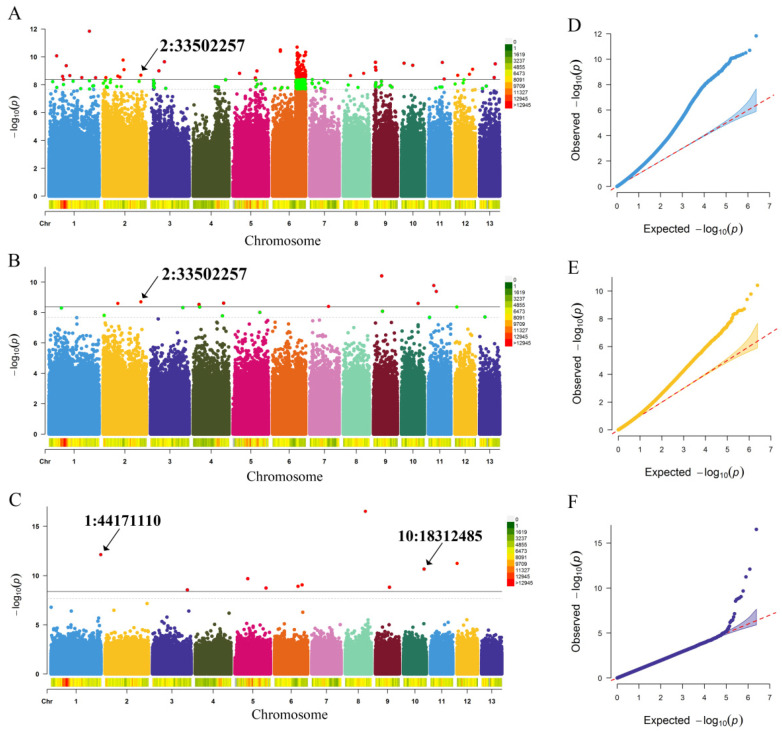
Genome-wide association study on the hundred-seed weight using three association analysis models. (**A**–**C**) The Manhattan plots of the GWAS on the hundred-seed weight using the MLM+K model, MLM+Q+K model, and FarmCPU model, respectively. The black solid line and dashed line indicate the significance threshold of 0.01 and 0.05, respectively. The spectrum column was used to represent the SNP density along 13 chromosomes of paper mulberry. (**D**–**F**) The QQ-plots of the GWAS on the hundred-seed weight using the MLM+K model, MLM+Q+K model, and FarmCPU model, respectively.

An association analysis of the hundred-seed weight was then performed based on the 2,414,978 high-quality SNPs using three association models. The mixed linear model with a kinship matrix (MLM+K model) detected 62 significant SNPs, which distributed on chromosomes 1, 2, 3, 5, 6, 8, 9, 10, 11, 12 and 13 (Figure 3A, Table S5). Using the mixed linear model with the Q-matrix and K-matrix (MLM+Q+K model), nine significant signals distributed on chromosomes 2, 4, 7, 9, 10 and 11 were detected (Figure 3B, Table S6). The FarmCPU model identified 10 significant signals, which distributed on chromosomes 1, 3, 5, 6, 9, 10 and 12 (Figure 3C, Table S7). Among them, the significant SNPs 2:13066816 and 2:33502257 were detected by both the MLM+K model and the MLM+Q+K model, and the significant SNP 6:24458586 was detected by both the MLM+K model and the FarmCPU model.

### 2.3. Candidate Gene and Genetic Variation Analysis

Based on the LD (linkage disequilibrium) decay distance of the paper mulberry population [31], the associated genes were screened within ~10 kb upstream and downstream of the significant SNPs. Through the MLM+K, MLM+Q+K and FarmCPU models, a total of 148, 18 and 12 associated genes were detected, respectively (Tables S5–S7). Among them, seven genes were identified through the common significant SNPs 2:13066816, 2:33502257 and 6:24458586. The candidate gene *Bp02g0551* was associated with the significant SNP 2:13066816, which encoded a retrotransposon integrase-like protein. Through the significant SNP 2:33502257, four candidate genes (*Bp02g2120*, *Bp02g2121*, *Bp02g2122*, *Bp02g2123*) were identified, which encoded the late embryogenesis abundant protein, leucine-rich repeat receptor-like protein kinase, LOB domain-containing protein and fatty acid desaturase, respectively. The candidate genes *Bp06g1794* and *Bp06g1795* were associated with the significant SNP 6:24458586, which encoded the reverse transcriptase and AAA-ATPase, respectively.

**Figure 4:**
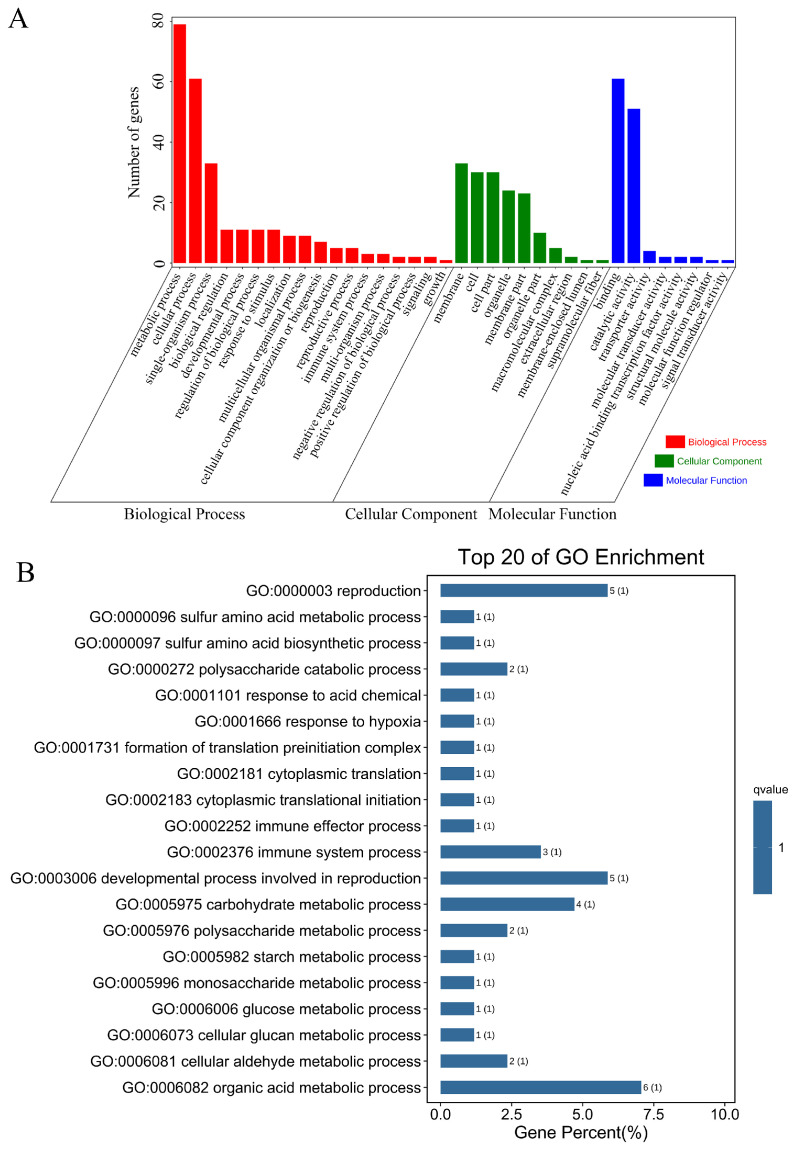
The GO annotation of the associated genes identified by the GWAS of hundred-seed weight through multiple models. (**A**) The Gene Ontology (GO) classification of the associated genes. (**B**) The top 20 GO terms in biological processes.

Then, the Gene Ontology (GO) and KEGG enrichment analysis were used to classify the function of the associated genes. We found that all the associated genes were classified into 36 GO terms, and the genes related to the “metabolic process”, “cellular process”, “binding”, “catalytic activity”, “membrane” and “cell” were comparatively more (Figure 4A). The top three GO terms in biological processes were the “organic acid metabolic process”, “developmental process involved in reproduction” and “reproduction”, which indicate that the related genes might be involved in regulating the seed development of paper mulberry (Figure 4B). In addition, the KEGG enrichment analysis exhibited that the “metabolic pathways” and “ubiquinone and other terpenoid-quinone biosynthesis” were the significant enriched pathways (Figure S1).

**Figure 5:**
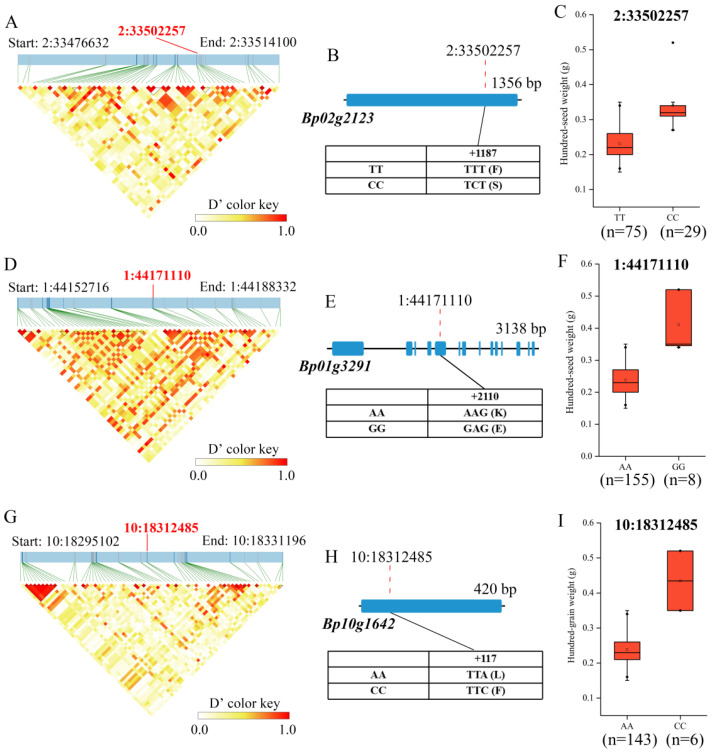
The analysis of the SNPs 2:33502257, 1:44171110 and 10:18312485. (**A**) The linkage disequilibrium heat map surrounding the SNP 2:33502257. (**B**) The gene structure of *Bp02g2123* and the DNA polymorphism of the nonsynonymous SNP 2:33502257. (**C**) Comparison of the hundred-seed weight of the individuals carrying different genotypes of 2:33502257. (**D**) The linkage disequilibrium heat map surrounding the SNP 1:44171110. (**E**) The gene structure of *Bp01g3291* and the DNA polymorphism of the nonsynonymous SNP 1:44171110. (**F**) Comparison of the hundred-seed weight of the individuals carrying different genotypes of 1:44171110. (**G**) The linkage disequilibrium heat map surrounding the SNP 10:18312485. (**H**) The gene structure of *Bp10g1642* and the DNA polymorphism of the nonsynonymous SNP 10:18312485. (**I**) Comparison of the hundred-seed weight of the individuals carrying different genotypes of 10:18312485.

Importantly, 25 candidate genes were directly hit by the significant SNPs, which encoded the fatty acid desaturase (*Bp02g2123*, *Bp06g1740*), serine/threonine-protein kinase (*Bp06g1907*), L-type lectin-domain containing receptor kinase (*Bp06g1785*), WRKY transcription factor (*Bp05g0302*), and F-box protein (*Bp10g1642*) (Table 1). Furthermore, the haplotypes of the significant SNPs located in the 25 candidate genes were analyzed. Among them, three important genetic variations in the coding regions of candidate genes (*Bp02g2123*, *Bp01g3291* and *Bp10g1642*) were identified, and the significant differences in hundred-seed weight were detected among the individuals carrying different genotypes (Figure 5). Because of the 1187nd base of *Bp02g2123* changing from T to C, the encoded amino acid changed from phenylalanine (F) to serine (S) (Figure 5B). We found that the paper mulberry individuals carrying 2:33502257-CC had higher hundred-seed weight than the individuals carrying 2:33502257-TT (Figure 5C). The significant SNP 1:44171110 located in the fifth exon of the candidate gene *Bp01g3291* (Figure 5E) encoded a phosphatidylinositol 4-kinase. The mutation of the 2210nd base (A to G) of *Bp01g3291* changed the encoded amino acid (lysine to glutamine) (Figure 5E), and the hundred-seed weight of paper mulberry individuals with 1:44171110-GG was significantly higher than the individuals carrying 1:44171110-AA (Figure 5F). The significant SNP 10:18312485 was also located in the exon region of the candidate gene *Bp10g1642,* encoding a F-box/kelch-repeat protein. The 117nd base of *Bp10g1642* changed from A to C and the encoded amino acid changed from leucine (L) to phenylalanine (F) (Figure 5H). Further analysis revealed that the paper mulberry individuals carrying 10:18312485-CC had higher hundred-seed weight than the individuals carrying 10:18312485-AA (Figure 5I). Therefore, these results indicated that the three genetic variations were significantly related to the hundred-seed weight of paper mulberry and might directly affect the function of the candidate genes.

Considering that the local temperature might be the main selective pressure for the natural variation of seed weight in woody plants, we further analyzed the expression patterns of associated genes under cold stress. The RNA-seq data suggested that some of the associated genes were responsive to cold stress (Figure S2, Table S8), such as *Bp02g2120* encoding a late embryogenesis abundant protein, *Bp03g1311* encoding a chlorophyll A-B binding protein, and *Bp12g0031* and *Bp12g0032* encoding the auxin responsive proteins. The predicted protein-protein interaction network indicated that the associated genes were closely related to each other, which might work together to regulate the seed weight of paper mulberry (Figure S3). In addition, the expression level of *Bp02g2123* was significantly up-regulated under cold stress, the expression level of *Bp01g3291* did not change, while *Bp10g1642* was not expressed (Figure S4), which indicated that *Bp02g2123* might be involved in the local temperature adaptation of woody plants.

### 2.4. Genome-Wide Analysis of the Fatty Acid Desaturase Genes 

Because *Bp02g2123* was identified by both the MLM+K model and the MLM+Q+K model, the variation of the significant SNP 2:33502257 in the coding region of *Bp02g2123* was closely associated with the hundred-seed weight, and *Bp02g2123* was significantly up-regulated by the low temperature, we inferred that *Bp02g2123* might directly affect the seed weight and local adaptation of paper mulberry. To better understanding the function of the candidate gene *Bp02g2123*, the genome-wide analysis of the *FAD* gene family in paper mulberry was performed, and 17 *BpFAD* genes were identified using the HMM search and BLAST alignment (Table S9). The length of BpFAD proteins ranged from 208 to 299 amino acids, and the molecular weight ranged from 23.11 to 33.47 kDa (Table S10).

**Figure 6:**
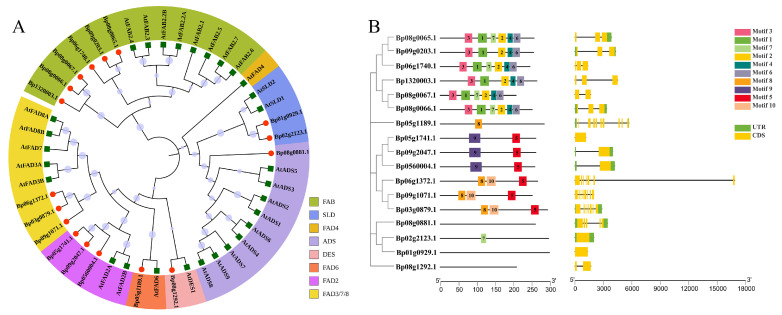
The analysis of the phylogenetic relationship, conserved motifs and gene structures of *FAD* family genes in paper mulberry. (**A**) The phylogenetic relationship of FAD proteins from paper mulberry (red filled circle) and *Arabidopsis* (green filled square). The neighbor-joining tree was constructed through the MEGA X program with 1000 bootstraps. (**B**) The analysis of the motifs and gene structures of the *FAD* family genes in paper mulberry. The conserved motifs were analyzed using the online analysis tool MEME.

In order to classify the *BpFAD* family genes and explore the phylogenetic relationship of the *FAD* family in paper mulberry, a phylogenetic tree was constructed using the full-length amino acid sequences of each FAD protein from paper mulberry and *Arabidopsis*. According to the classification results in *Arabidopsis*, the *BpFAD* genes were divided into eight categories (Figure 6A). The analysis of the sequences indicated that the close *FAD* genes had similar gene structures (Figure 6B). Ten conserved motifs of BpFAD proteins were then identified through MEME (Figure S5), and the results showed that the BpFAD proteins from the same categories had similar motif distributions (Figure 6B). Motif 1, 2, 3, 4 and 6 only occurred in the FAB proteins, motif 9 only occurred in the FAD2 proteins, and *Bp02g2123* only had motif 7 (Figure 6B).

Based on the genome annotation file of paper mulberry, the chromosome distributions of *BpFAD* genes were displayed. The result showed that 15 *BpFAD* genes were located in seven chromosomes, while two *BpFAD* genes were located in two scaffolds; among them, five *BpFAD* genes were mapped on chromosome 8 (chr08) (Figure S6). Three gene pairs having segmental duplication events were identified: *Bp06g1372*/*Bp09g1071*, *Bp06g1372*/*Bp03g0879* and *Bp09g1071*/*Bp03g0879* (Figure S7), and all of these three genes belonged to the same sub-clade. Furthermore, five paper mulberry *FAD* genes had collinearity relationships with six *Arabidopsis FAD* genes (Figure S8).

**Figure 7:**
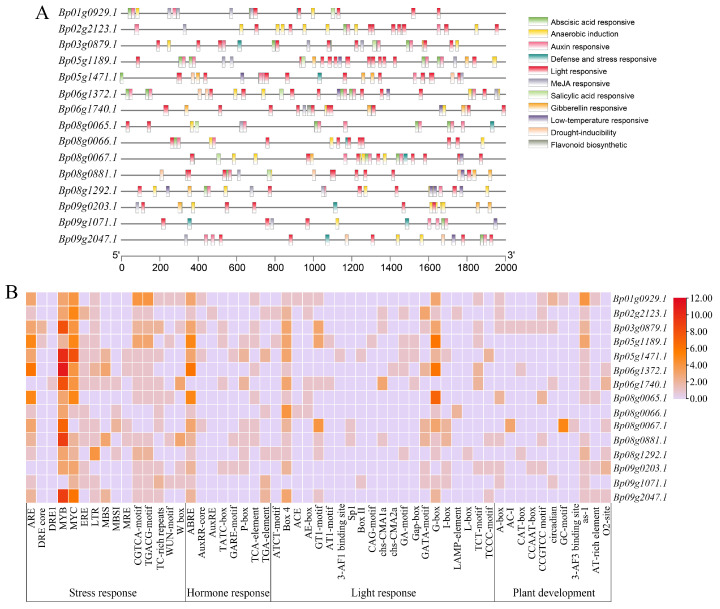
The analysis of the *cis*-acting regulatory elements of *FAD* family genes in paper mulberry. (**A**) The location of the *cis*-acting regulatory elements in the upstream 2000-bp of the *BpFADs*, which were identified through the online analysis tool PlantCARE. (**B**) The number of the *cis*-acting regulatory elements in the promoter regions of *BpFAD* genes. The color scale represents the number of each *cis* element in every *BpFAD* gene.

A *cis*-regulatory element analysis on the promoter regions of *FAD* genes in paper mulberry was performed, and the results indicated that there were a number of stress response elements, hormone response elements and light response elements in the promoter regions of *BpFAD* genes (Figure 7). We found that the promoter regions of almost all *BpFAD* genes contained many MYB and MYC elements (Figure 7B), and the abscisic acid-responsive elements also existed in almost all *BpFAD* genes, while auxin responsive elements, gibberellin responsive elements, Me-JA responsive elements and salicylic acid responsive elements also were widespread in the promoter regions of *BpFAD* genes. In addition, some low-temperature responsive elements (LTRs) in the promoter regions of *BpFAD* genes were also identified. These results suggested that *BpFAD* genes might play an important role in stress response and the hormonal pathways.

### 2.5. Expression Pattern Analysis of BpFAD Genes 

**Figure 8:**
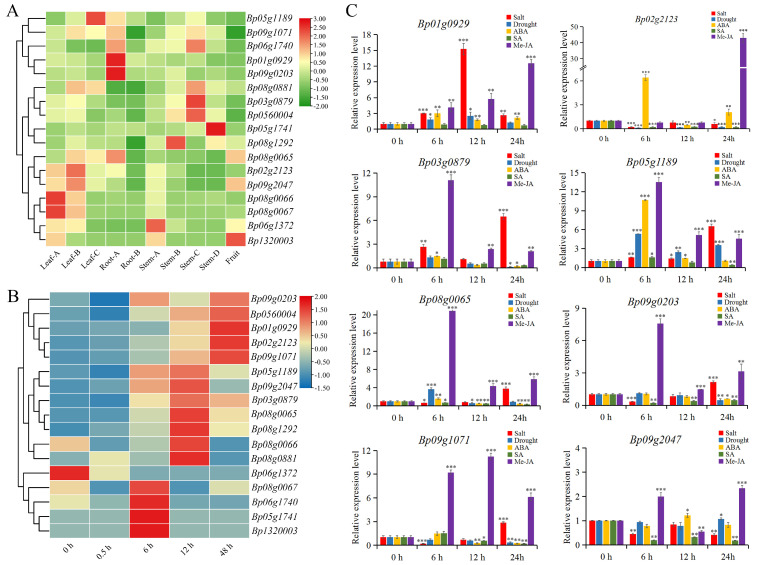
The expression profiles of *BpFAD* family genes. (**A**) The expression patterns of *BpFAD* genes in different tissues based on the FPKM values. The transcript levels of *BpFAD* genes were shown through the color gradient; green to red represents the transcript levels from low to high. Leaf-A represents the young leaf; Leaf-B represents the developing leaf; Leaf-C represents the climax leaf; Root-A represents the root tip; Root-B represents the taproot; Stem-A represents the apical bud; Stem-B represents the immature stem; Stem-C represents the partially lignified stem; Stem-D represents the mature stem. (**B**) The expression profiles of *BpFAD* genes under 4 °C treated with different times based on the FPKM values; blue to red represents the transcript levels from low to high. (**C**) The expression patterns of *BpFAD* genes under abiotic stresses and hormone treatments. A quantitative RT-PCR was used to explore the expression levels of *BpFAD* genes. *BpGAPDH* was selected as an internal control. Salt: leaves treated with 250 mM NaCl. Drought: leaves treated with 20% PEG6000. ABA: leaves treated with 100 µM ABA. SA: leaves treated with 100 µM SA. Me-JA: leaves treated with 100 µM Me-JA. * represents *p <* 0.05, ** represents *p <* 0.01, *** represents *p <* 0.001.

To further understand the possible function of *BpFAD* genes, the expression patterns of *BpFADs* were analyzed using the RNA-seq data from ten different tissues, including roots, stems, leaves and fruits. The results indicated that the expression patterns of *BpFADs* in different tissues were diverse, and the expression of some genes showed high specificity in certain tissues (Figure 8A, Table S11). For example, *Bp01g0929* and *Bp09g0203* had high expression levels in root tips, *Bp08g0066* and *Bp08g0067* were highly expressed in young leaves, while *Bp03g0879* and *Bp05g1741* exhibited higher expression levels in stems. Furthermore, *Bp02g2123* was relatively highly expressed in leaves, especially in developing ones. The significant tissue specificity implied that *BpFAD* genes might have different functions during the development of the plant.

The expression pattern of *BpFAD* genes in response to cold stress was also analyzed using the RNA-seq data. The results reflected that the majority of *BpFAD* genes were significantly up-regulated under cold stress (Figure 8B, Table S12), not just *Bp02g2123* mentioned earlier. Among them, *Bp01g0929* and *Bp02g2123* reached the highest expression levels at the low temperature treatment for 48 h, which belonged to the same *SLD* sub-clade. The highest expression levels of *Bp03g0879*, *Bp05g1189*, *Bp08g0065*, *Bp08g0881* and *Bp09g2047* were exhibited at low temperature treatment for 12 h. These results reflected that different *BpFAD* genes might play the main roles during different stages of cold stress. 

Considering that the promoter regions of *BpFAD* genes contain a large number of regulatory elements about the stress response and hormonal regulation, the relative expression levels of 10 selected *BpFADs* under other different abiotic stresses also were explored through a quantitative real-time PCR (qRT-PCR), including drought stress, salt stress and three hormone treatments (ABA, Me-JA and SA). The results showed that the expression levels of eight *BpFAD* genes were significantly changed during the phytohormone and stress treatments (Figure 8C), except for *Bp05g1741* and *Bp08g1292*. Salt stress could significantly induce the expression of *Bp01g0929*, *Bp03g0879, Bp05g1189, Bp08g0065, Bp09g0203* and *Bp09g1071*. Among them, *Bp01g0929* was highly induced at 12 h and then decreased, while the other genes were highly induced at 24 h. Only *Bp05g1189* and *Bp08g0065* were significantly up-regulated under drought stress, while *Bp02g2123*, *Bp09g0203* and *Bp09g1071* were significantly down-regulated under drought stress, and the expression levels of other *BpFAD* genes were not clearly altered. Interestingly, almost all of the *BpFAD* genes were significantly up-regulated under Me-JA treatments. The expression of *Bp03g0879*, *Bp05g1189*, *Bp08g0065*, *Bp09g0203* reached the highest expression levels at 6 h, while *Bp01g0929, Bp02g2123* and *Bp09g2047* showed the highest expression levels at 24 h and *Bp09g1071* reached the highest expression level at 12 h. ABA treatments significantly induced the expression of *Bp02g2123* and *Bp05g1189*, while the expression of *Bp02g2123*, *Bp05g1189*, *Bp08g0065*, *Bp09g2023, Bp09g1071* and *Bp09g2047* were significantly repressed by SA treatments. In general, *BpFAD* genes responded to various stresses and hormone treatments and might play important roles in environmental adaptation and plant development.

## 3. Discussion

Widely distributed plants usually adapt to different environments through phenotypic variations [33], and woody plants also adapt to the changeable environments through regulating seed traits [10]. In this study, the seeds of paper mulberry were collected from 19° N to 41° N, and the hundred-seed weight ranged from 0.15 g to 0.52 g, which showed a significant variation along the geographic gradient. We found that the hundred-seed weight of paper mulberry was increased from low latitudes to high latitudes along the latitudinal-temperature gradient, which was consistent with some previous studies [9,11]. This study has shown that plants from higher latitudes will produce larger seeds in *Acer platanoides* [9]. Using 11 forest herb species, previous research found that there is a positive correlation between the seed mass and latitude within species [34]. A recent study also reported that the seed size increases from low latitudes to high latitudes in invasive plants [11]. Conversely, some studies also found that the plants from low latitudes tend to produce larger seeds [10,35], which suggest that there may be different mechanisms of the local environment adaptation in plants. Climate factors could also affect the seed size or seed weight, such as temperature and precipitation [36]. The previous study in *A. platanoides* found that the plants under colder conditions produce heavier seeds than the plants under warmer environments [9]. In the present study, we also found that the hundred-seed weight of paper mulberry was associated with climate factors, especially the mean temperature, active accumulated temperature, extreme low temperature, and frost-free period of the sampling sites. Therefore, we inferred that the natural variation of seed size or seed weight along the latitudinal-temperature gradient should be a crucial adaptive strategy of woody plants. 

To better understand the underlying genetic basis of seed weight in natural woody populations, the genome-wide association analysis of hundred-seed weight was performed in paper mulberry. Through three different models, a series of associated genes were detected, including the E3 ubiquitin ligase, protein phosphatase, and auxin responsive protein. The ubiquitin-proteasome pathway is essential for seed development [37], and a previous study found that *GRAIN WEIGHT 2* (*GW2*) encoding an E3 ubiquitin ligase can regulate grain width and weight [16]; further studies in rice reveal that the GW2 protein can ubiquitinate the WG1 (WIDE GRAIN 1) protein and control the grain weight and grain size through the GW2-WG1-OsbZIP47 regulatory module [38]. The protein phosphatase is involved in regulating seed weight, and the *PP2C-1* allele can increase the seed weight in soybean [21]. Furthermore, the overexpression of *auxin response factor 19* (*ARF19*) can increase the seed size and seed weight of woody plants [23], indicating that the auxin pathway plays an important role in seed development. In the current study, 25 candidate genes were directly hit by the significant SNPs, and the related genes encoded the L-type lectin-domain containing receptor kinase, WRKY transcription factor, serine/threonine-protein kinase, and F-box protein. Interestingly, the GWAS analysis on seed size in watermelon also identified candidate genes encoding the auxin-responsive protein, F-box protein, receptor kinase and protein phosphatase [28]. The related studies report that WRKY transcription factors correlate to the seed size in wild soybean [39] and foxtail millet [40]. The photoperiod can also influence the seed weight and yield of plants [41], and some associated genes involved in light harvesting and responding also were identified in this study, such as the chlorophyll a/b binding protein. Chlorophyll a/b binding proteins are important components of the light-harvesting complex of photosystem II (PSII) [42], which are tightly regulated by multiple environmental signals, especially light signals [43]. A previous study also showed that the seed germination and post-germination growth are positively regulated by chlorophyll a/b-binding family members in response to ABA [43]. Thus, we put forward that the associated genes identified in this study may play a major role in the regulation of seed development in paper mulberry.

In the current study, three crucial genetic variations responsible for seed weight were identified, which were located in the coding regions of candidate genes and are significantly associated with the hundred-seed weight (Figure 5). Among them, *Bp10g1642* encoding an F-box protein and the paper mulberry individuals carrying 10:18312485-CC had higher hundred-seed weight than the individuals carrying 10:18312485-AA. The previous study revealed that the F-box protein is a key regulator of organ sizes in *Medicago truncatula*, which is valuable for increasing the seed size and yield [44]. The previous research has reported that *OsFBK12* (an F-box protein) is also involved in regulating the seed size and grain number of rice [45]. The significant SNP 2:33502257 was located in the exon region of the *FAD* gene *Bp02g2123*, and the hundred-seed weight of paper mulberry individuals with 2:33502257-CC was higher than the individuals carrying 2:33502257-TT. The fatty acid desaturase is a key enzyme in the synthesis of unsaturated fatty acids [46], which is critical for the seed development and directly affects the seed weight of plants [47]. The study has shown that miR167A-CsARF8 regulates the expression of *fatty acid desaturase 3* (*CsFAD3*), which can affect the seed size of camelina (*Camelina sativa*) [47]. and the seeds of *GmFAD3*-silenced plants are larger and heavier in soybean [48]. The seed weight and oil content of *CsaFAD7* and *CsaFAD8* transgenic lines are decreased in *Arabidopsis thaliana*, and the result shows that *FAD* genes could affect the seed morphology [49]. Previous studies have shown that cold-tolerant plants contain higher levels of unsaturated fatty acids, and *FAD* is involved in the cold stress response [50]. Most interestingly, the expression level of *Bp02g2123* was significantly up-regulated under cold stress. The previous study reported that the seeds from higher latitudes have larger seed size and more fatty acids, which may be a strategy for adapting to the local cold weather [11]. Therefore, we speculate that the genetic variations in the coding regions of candidate genes may directly affect the gene functions and seed weight of paper mulberry, thus helping individuals adapt to the local climate.

As plant growth and seed development are significantly affected by environmental stresses, the variation of seed traits may be the result of local environmental adaptations. The fatty acid desaturase is involved in response to various biotic and abiotic stresses, which also plays crucial roles in the seed development and biosynthesis of jasmonic acid (JA) in plants [48]. The study in sunflower has shown that *HaFAD* genes significantly change their expression levels under biotic and abiotic stresses [51], while the expression level of *FAD* genes and the activity of fatty acid desaturase are also changed under salt stress in peanut [52]. The overexpression of *LeFAD3* improves the chilling tolerance of tomato [53], and the overexpression of *GmFAD3A* can enhance the cold tolerance and seed germination rate of rice [54]. In the present study, a large number of regulatory elements about the stress response and hormonal regulation were identified in the promoter regions of *BpFAD* genes (Figure 7), which suggested that *BpFAD* genes might be involved in responding to different environmental stresses. The RNA-seq data and qRT-PCR results demonstrated that *BpFAD* genes could be induced by cold stress, salt stress, drought stress and hormone treatments. Jasmonic acid plays an important role in stress response and seed development [55]. In the current study, most of the *BpFAD* genes were significantly up-regulated under Me-JA treatments, especially the crucial candidate gene *Bp02g2123*, indicating that *BpFAD* genes might be involved in the JA signaling pathway as JA-responsive genes. Transcription factors (TFs) also play important roles in modulating the biosynthesis of fatty acids through regulating the transcription of *FAD* genes, such as *bHLH* [56], *bZIP* [57], and *MYB* [58]. In addition, the previous study shows that MaMYB4 can repress the transcription of *MaFADs* in cold stress [59], while the accumulation and composition of fatty acids are also regulated by MYB transcription factors during seed development [60]. Interestingly, almost all *BpFAD* genes contained many MYB and MYC elements in their promoter regions, thus we inferred that *BpFAD* genes might also be regulated by MYB or MYC transcription factors. Taken together, the *FAD* gene family in paper mulberry might be closely related to the seed development and stress response, and future studies should be focused on the specific molecular mechanisms of how *BpFAD* genes regulate the seed weight and environmental adaptation of paper mulberry.

## 4. Materials and Methods

### 4.1. Samples Collection and Hundred-Seed Weight Analysis

The experimental materials included 198 wild paper mulberry individuals which were collected from different geographic regions, including 40 individuals which were reported in a previous study [31]. The mature fruits were collected from the paper mulberry plants, and the seeds were washed out and dried naturally and were then used for counting the hundred-seed weight. In this study, the seeds of more than three paper mulberry plants from the same sample sites were collected, and the hundred-seed weight of the seeds from the same plants was counted three times, and the average of the replicates was defined as the final hundred-seed weight data for GWAS analysis. Meanwhile, the leaves were stored at −80 °C and used for extracting genomic DNA. The mean values of the bioclimatic variables of the sampling sites in the past thirty years were accessed from http://data.cma.cn (accessed on 17 November 2017), including the mean temperature, extreme low temperature, active accumulated temperature, frost-free period, annual precipitation, and sunshine hours. The frequency distribution of hundred-seed weight, as well as the linear fitting between the hundred-seed weight and bioclimatic variables were then analyzed.

### 4.2. DNA Extraction and Whole Genome Re-Sequencing

The total genomic DNA was extracted from the leaves stored at −80 °C using a kit (Tiangen, Beijing, China) following the manufacturer’s protocol. After testing for the quality and quantity of DNA, the NEB Next Ultra DNA Library Prep Kit (NEB, MA, USA) was used for preparing sequencing libraries. The qualified libraries were then sequenced using the Illumina HiSeq X Ten platform. And the sequencing data can be found in the NCBI database with the BioProject codes (PRJNA870972 and PRJNA635453). The quality filtering and SNP calling were performed as the previous study [31]. Finally, a total of 2,414,978 SNPs with missing rate <50% and minor allele frequency (MAF) >0.05 were used for the following association analysis.

### 4.3. Genome-Wide Association Study (GWAS)

For association analysis, the mixed linear model (MLM) was used to perform the analysis with the TASSEL V3.0 software [61]. In the MLM association models, we used two methods (MLM+K and MLM+Q+K), while the MLM+K model took a kinship matrix into account, and the MLM+Q+K model took both the K-matrix and Q-matrices into account. The fixed and random model Circulating Probability Unification (FarmCPU) package also was used to carry out the association analysis [62]. A Bonferroni test was used to estimate the whole-genome significance threshold, which was set as 0.01/total SNPs (−log_10_(*p*) = 8.38). Finally, the R software package was used to generate the Manhattan plots and QQ-plots [63]. 

### 4.4. Candidate Gene and Genetic Variant Analysis

According to the LD decay distance of paper mulberry [31], candidate genes were detected in the upstream and downstream ~10 kb range of significant SNPs. In order to understand the function of candidate genes, the functional enrichment analysis and gene ontology (GO) term enrichment were performed using the AgriGO analysis toolkit and KEGG database. LD blocks of important SNPs were created using the LDBlockShow program [64]. The protein-protein interaction analysis of candidate genes was performed using the online database STRING (https://cn.string-db.org/), and Cytoscape (version 3.9.1) software was used to display the interaction network [65]. The heat map of expression levels of candidate genes under cold stress (4 °C treatment with 0 h, 0.5 h, 6 h, 12 h, 48 h) was created using the transcriptome data [66].

### 4.5. Identification of BpFAD Genes and Phylogenetic Analysis

To identify *FAD* family genes in paper mulberry, we downloaded the HMM file for the FAD domain (PF00487) from the Pfam database (http://Pfam.xfam.org/) [67], and then the HMMER 3.2 was used to identify the potential *FAD* genes from the paper mulberry genome [68]. Meanwhile, the genome data of *Arabidopsis* was downloaded from the Ensembl Plants database (http://plants.ensembl.org/), and the FAD protein sequences in *Arabidopsis* were picked out according to a previous study [69], which was used as queries to search the *FADs* of paper mulberry with an E value < 1 × 10^−10^ in the BLASTp. The potential *FAD* genes identified through the above two methods were further verified using the SMART and Pfam databases [67,70]. To classify the *FAD* members in paper mulberry, the MEGA X was used to construct the phylogenetic tree using the neighbor-joining (NJ) method with a bootstrap value of 1000 [71]. The online tool iTOL (https://itol.embl.de/) was then used to color the phylogenetic tree [72], and the *FAD* members in paper mulberry were classified referring to the classification results of *Arabidopsis* [69]. 

The physicochemical properties of *BpFADs* were analyzed through the online program ExPASy (https://www.expasy.org/), and the online program MEME (version 5.4.1, https://meme-suite.org/meme/) was used to identify the conserved motifs of the *BpFAD* gene family. We then extracted the 2000bp sequences upstream of the coding region of *BpFAD* genes, and the *cis*-acting elements analysis of promoters was performed using the online program PlantCARE (http://bioinformatics.psb.ugent.be/webtools/plantcare/html/). Next, the above results, chromosome locations and exon/intron structures of each *BpFAD* gene were visualized using the TBtools software [73]. The TBtools software was also used to investigate the duplication events of the *BpFAD* gene family and the collinearity relationships for *FAD* genes between paper mulberry and *Arabidopsis* using the BLASTp and MCScanX methods [74].

### 4.6. Gene Expression Analysis

Based on the transcriptome data of different paper mulberry tissues (leaf, stem, root and fruit) and the transcriptome data under cold stress (4 °C treatment with 0 h, 0.5 h, 6 h, 12 h, 48 h), which were published in previous studies [66,75], the expression patterns of the *BpFAD* gene family were investigated. The FPKM values of each *BpFAD* gene were extracted, and the program TBtools was used to make the heatmaps. The related FPKM values are listed in Tables S11 and S12.

The qRT-PCR (quantitative real-time PCR) was then used to explore the expression patterns of *BpFAD* genes under different abiotic stresses. The plantlets of paper mulberry were treated with 250 mM NaCl, 20% PEG6000, 100 µM ABA, 100 µM SA and 100 µM Me-JA for 0 h, 6 h, 12 h, and 24 h [76]. The second leaves fully unfolded and were collected, and total RNAs were extracted with a kit (TaKaRa, Beijing, China) following the manufacturer’s procedure. The cDNA was synthesized using a PrimeScript RT Reagent Kit (TaKaRa, Beijing, China), and the SYBR-Green PrimeScript RT-PCR Kit (Takara, Beijing, China) was used to conduct the qRT-PCR reactions according to the manufacturer’s instructions. Each independent biological replicate was performed in three technical replicates, and *BpGAPDH* in paper mulberry was selected as an internal control. Finally, the expression levels of each *BpFAD* gene were calculated through the 2^−^^△△Ct^ method [77]. The primers of each gene are listed in Table S13.

## 5. Conclusions

Our results suggested that the hundred-seed weight of paper mulberry was increased from south to north along the latitudinal-temperature gradient, which might be a crucial adaptive strategy of woody plants. The significant SNPs and candidate genes highlight the important underlying genetic basis of seed weight in woody plants. And the genetic variations in the coding regions might directly affect the function of candidate genes as well as the seed weight of paper mulberry. Furthermore, the genome-wide investigation and expression pattern analysis indicated that *BpFAD* genes were involved in response to various stresses, which was thought to influence the local adaptation of paper mulberry. To sum up, our findings provide valuable genetic variations responsible for seed weight, and will enhance the understanding of the genetic basis of seed weight in woody plants.

## Figures and Tables

**Table 1:** Summary of the candidate genes which were directly hit by the significant SNPs.

Chromosome	SNP	*p* Value	Candidate Gene	Start	End	Gene Function
chr01	41,331,575	3.1236 × 10^−9^	*Bp01g3013*	41,328,341	41,331,943	Glutamate/aspartate-prephenate aminotransferase
chr02	17,697,129	1.7214 × 10^−10^	*Bp02g0793*	17,685,050	17,701,778	Histone-lysine N-methyltransferase
chr02	33,502,257	2.0931 × 10^−9^	*Bp02g2123*	33,503,443	33,502,257	Fatty acid desaturase
chr03	7,395,071	1.0063 × 10^−9^	*Bp03g0908*	7,392,531	7,395,261	Coiled-coil domain-containing protein
chr05	5,767,053	1.5512 × 10^−9^	*Bp05g0302*	5,766,656	5,771,404	WRKY transcription factor
chr05	19,747,070	3.2621 × 10^−9^	*Bp05g1115*	19,746,216	19,747,481	Unknown
chr06	20,776,777	1.4767 × 10^−9^	*Bp06g1564*	20,776,626	20,777,048	Gypsy retrotransposon integrase-like protein
chr06	22,090,146	5.2352 × 10^−11^	*Bp06g1638*	22,090,053	22,090,610	KRAB-A domain-containing protein
chr06	23,468,712	2.5837 × 10^−9^	*Bp06g1740*	23,467,969	23,469,334	Fatty acid desaturase
chr06	24,234,952	5.1651 × 10^−10^	*Bp06g1785*	24,231,112	24,236,984	L-type lectin-domain containing receptor kinase
chr06	24,458,029	9.9772 × 10^−10^	*Bp06g1794*	24,451,588	24,458,586	Reverse transcriptase
chr06	25,389,813	3.84 × 10^−9^	*Bp06g1839*	25,385,775	25,392,659	Integrase-like protein
chr06	25,777,571	8.2208 × 10^−10^	*Bp06g1861*	25,776,161	25,779,520	Reverse transcriptase
chr06	26,694,446	3.7558 × 10^−10^	*Bp06g1907*	26,692,270	26,710,027	Serine/threonine-protein kinase
chr06	26,991,418	2.0725 × 10^−9^	*Bp06g1916*	26,990,342	26,992,820	Transposon Ty3-I Gag-Pol polyprotein
chr06	28,791,979	9.0384 × 10^−10^	*Bp06g2004*	28,791,621	28,792,100	Unknown
chr06	29,318,593	2.462 × 10^−9^	*Bp06g2035*	29,318,125	29,318,753	Unknown
chr08	6,440,358	2.2263 × 10^−9^	*Bp08g0810*	6,439,511	6,441,583	Protein SRG1
chr08	18,173,015	1.5181 × 10^−9^	*Bp08g1898*	18,172,259	18,185,337	Serine/threonine-protein phosphatase
chr09	2,133,099	8.9302 × 10^−10^	*Bp09g0165*	2,128,911	2,137,300	Histone-lysine N-methyltransferase
chr12	2,429,893	2.116 × 10^−9^	*Bp12g0266*	24,28,380	2,432,304	CTL-like protein
chr13	12,974,919	3.0993 × 10^−9^	*Bp13g0906*	12,972,842	12,981,872	Perakine reductase
chr01	44,171,110	4.56 × 10^−10^	*Bp01g3291*	44,165,116	44,176,839	Phosphatidylinositol 4-kinase
chr06	24,458,029	8.78 × 10^−10^	*Bp06g1794*	24,451,588	24,458,586	Reverse transcriptase
chr10	18,312,485	2.19 × 10^−11^	*Bp10g1642*	18,312,369	18,312,788	F-box/kelch-repeat protein

## Data Availability

The re-sequencing sequences underlying this study have been deposited in NCBI database under BioProject code: PRJNA870972 and PRJNA635453.

## Supplementary Materials

The following supporting information can be downloaded at: https://www.mdpi.com/article/10.3390/ijms232012520/s1.
