# Supplementary material for: Identification and Investigation of the Genetic Variations and Candidate Genes Responsible for Seed Weight via GWAS in Paper Mulberry
Source: Int J Mol Sci. 2022 Oct 19;23(20):12520. doi: 10.3390/ijms232012520 (PMC9604540; doi:10.3390/ijms232012520)
Supplement: Supplementary file 1 [file ijms-23-12520-s001.zip › Sup-figures.pdf]

Electronic Supplementary Materials

Identification and Investigation of the Genetic Variations and Candidate Genes Responsible for Seed Weight via GWAS in Paper Mulberry

Yanmin Hu, Xianjun Peng and Shihua Shen

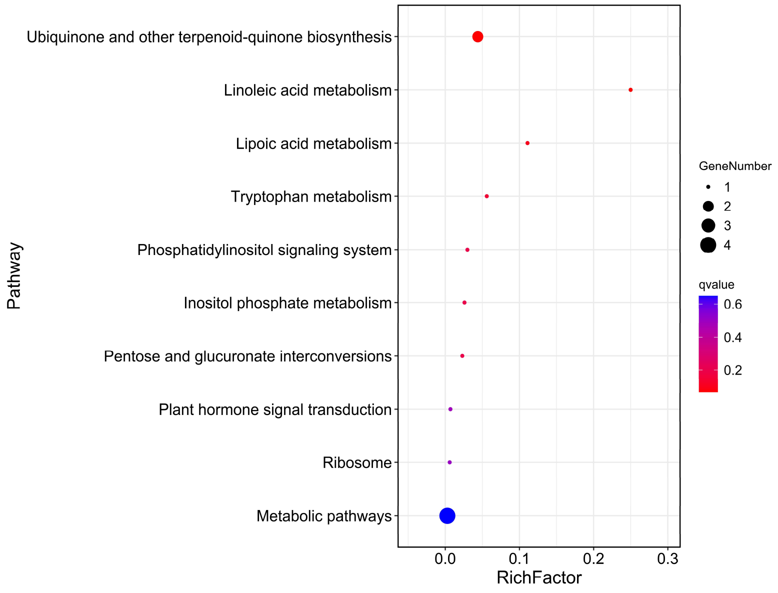

Figure S1. The KEGG annotation of the associated genes identified by GWAS of hundred-seed weight through multiple models.

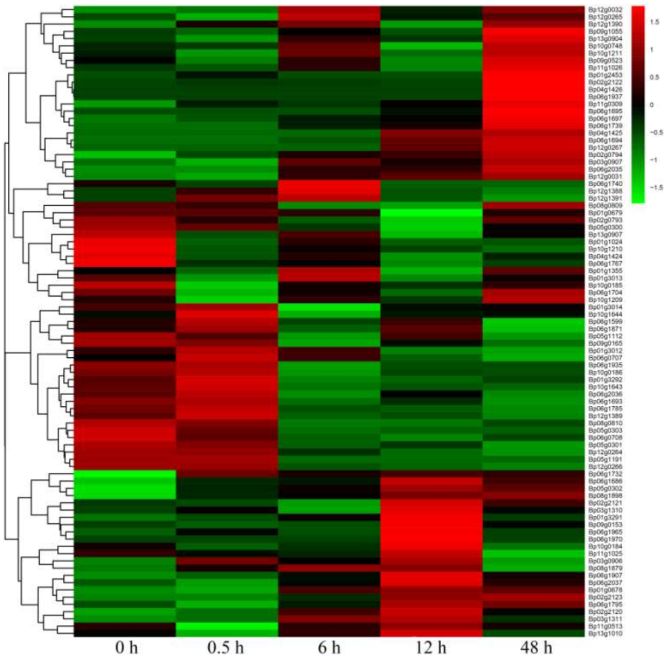

Figure S2. The heat map of the associated genes identified by GWAS of hundred-seed weight according to the transcriptome data under cold stress. 0 h, 0.5 h, 6 h, 12 h and 48 h represent 4℃ treated with different times. The graded color scale from green to red was used to display the transcript levels of the associated genes.

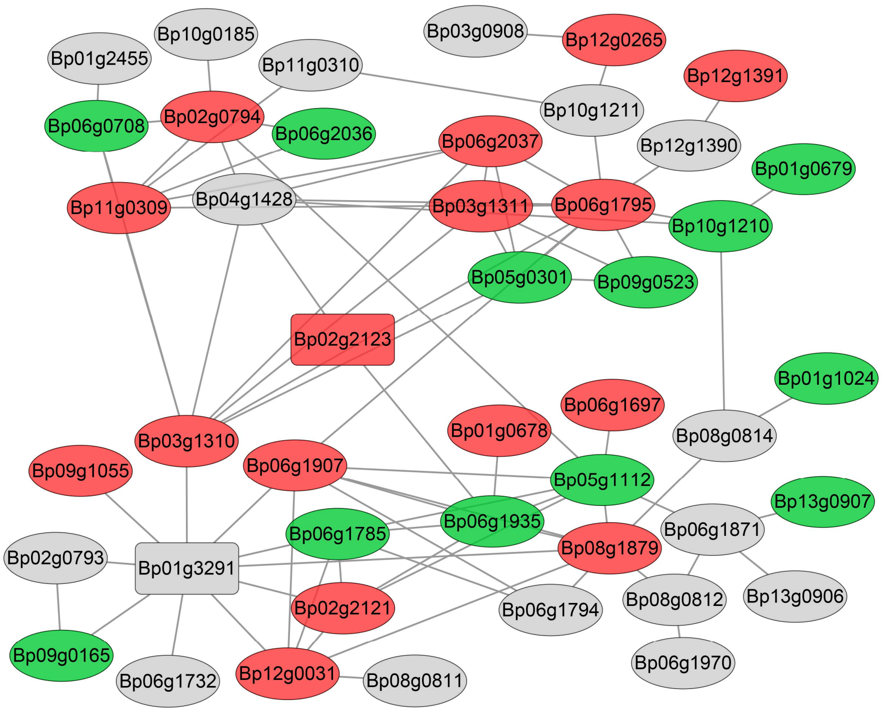

Figure S3. Protein-protein interaction analysis of the associated genes identified by GWAS of hundred-seed weight. The read color represents the related genes up-regulated under cold stress, the green color represents the related genes down-regulated under cold stress, and the gray color represents that the expression level of the related genes did not change under cold stress.

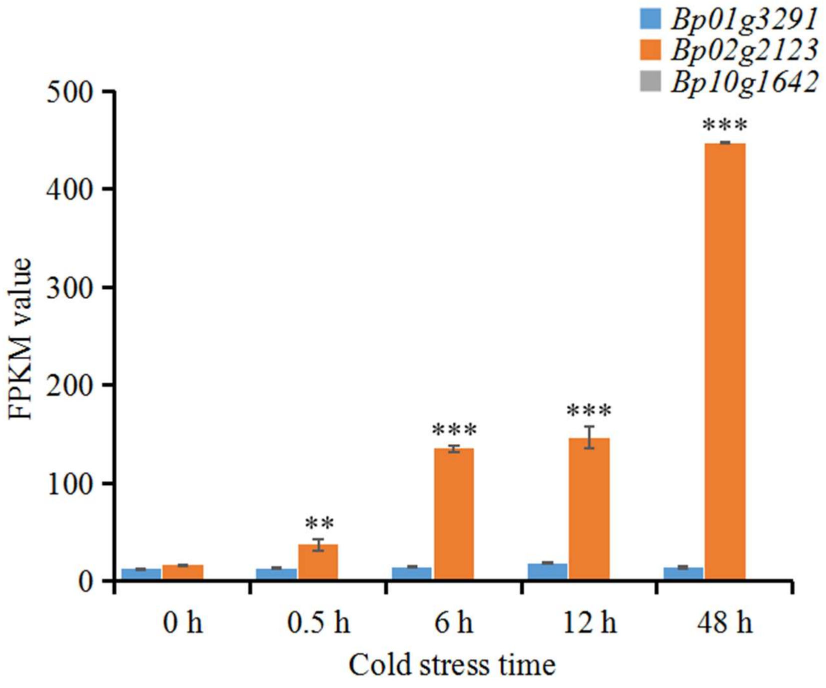

Figure S4. Expression patterns of *Bp01g3291*, *Bp02g2123* and *Bp10g1642* in paper mulberry under 4℃ treated with different times based on the FPKM values. 0 h, 0.5 h, 6 h, 12 h and 48 h represent 4℃ treated with different times. ** represents $p < 0.01$, *** represents $p < 0.001$.

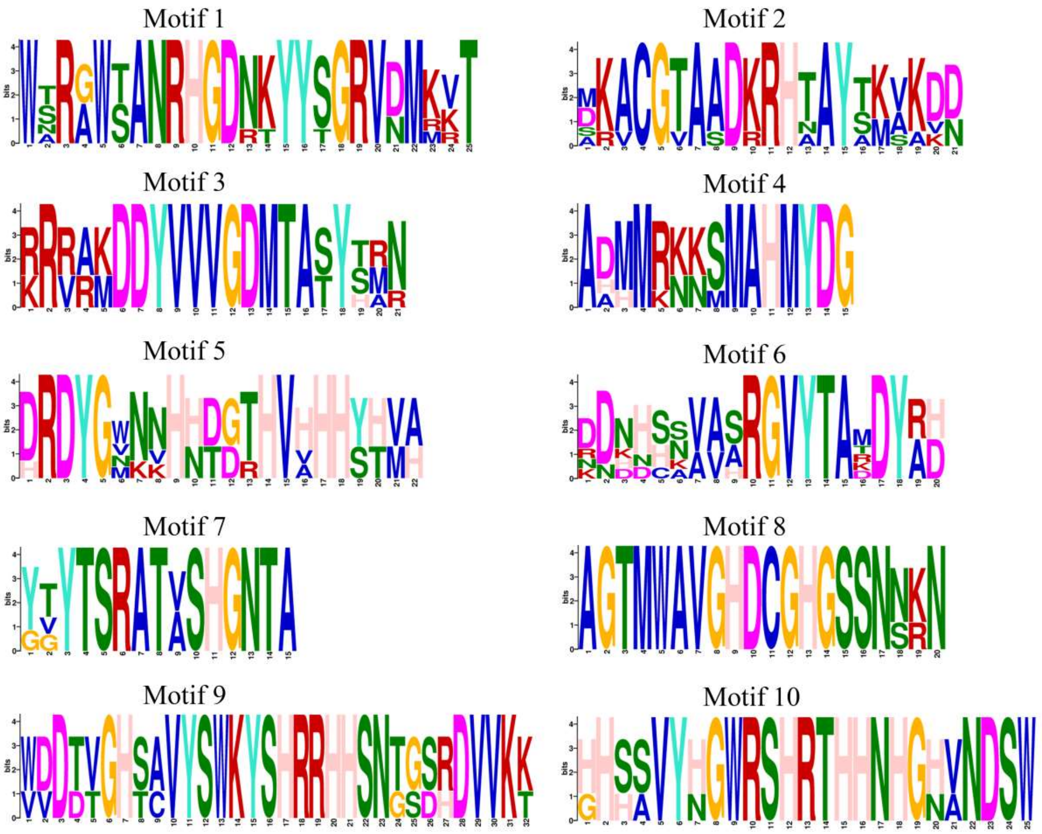

Figure S5. The ten conserved motifs of BpFAD proteins in paper mulberry, which were identified through the online analysis tool MEME (version 5.4.1).

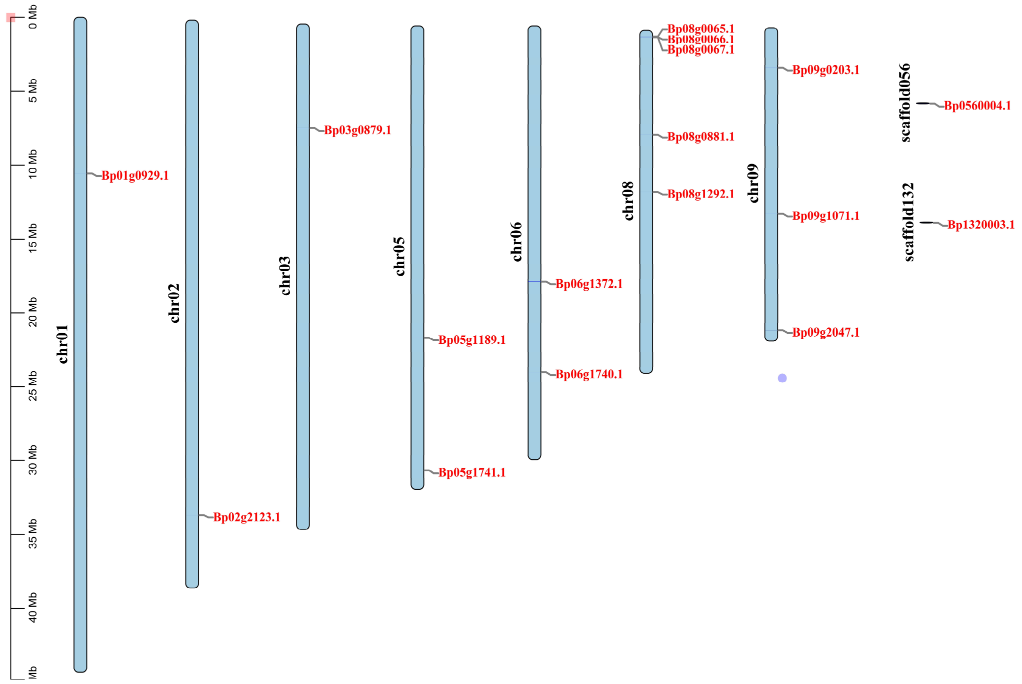

Figure S6. Chromosomal distribution of *FAD* genes from paper mulberry. Based on the paper mulberry genome data, the 17 *FAD* genes were mapped on 7 chromosomes and 2 scaffolds.

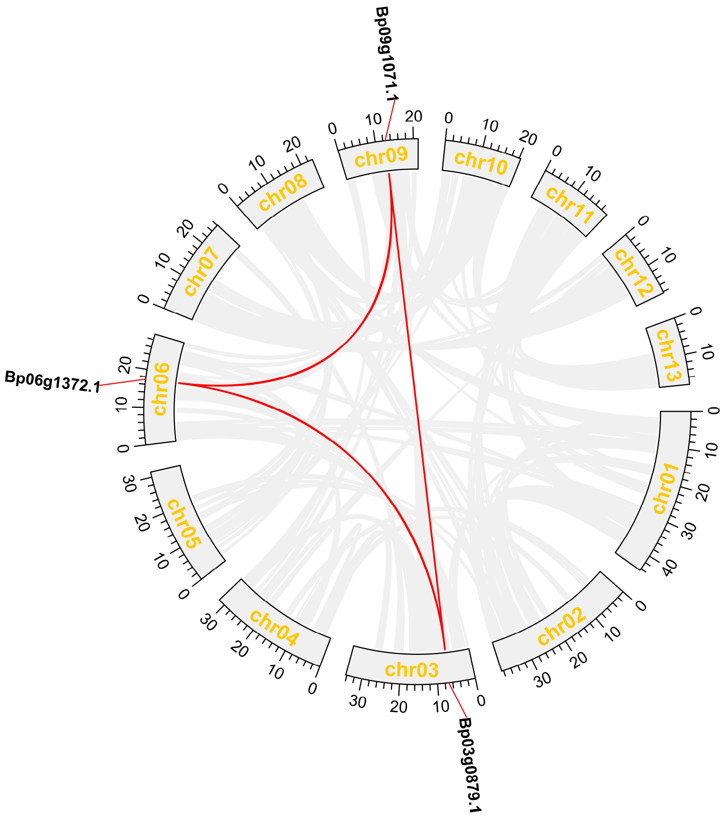

Figure S7. Duplication analysis of the *FAD* genes in paper mulberry, and the red lines represent the three pairs of duplicated genes.

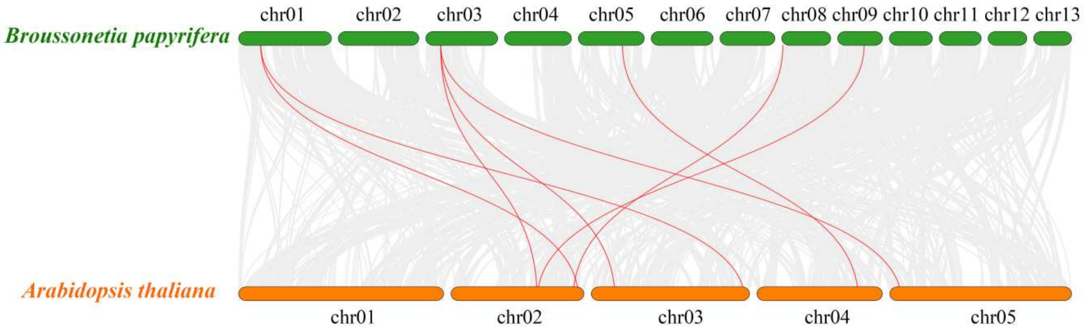

Figure S8. Collinearity analysis of the *FAD* genes in paper mulberry and *Arabidopsis*.
